# Circulating and synovial antibody profiling of juvenile arthritis patients by nucleic acid programmable protein arrays

**DOI:** 10.1186/ar3800

**Published:** 2012-04-17

**Authors:** David S Gibson, Ji Qiu, Eliseo A Mendoza, Kristi Barker, Madeleine E Rooney, Joshua LaBaer

**Affiliations:** 1 Arthritis Research Group, Centre for Infection and Immunity, Health Science Building, Queen's University of Belfast, 97 Lisburn Road, Belfast BT9 7BL, UK; 2 Division of Endocrinology, Metabolism and Diabetes, School of Medicine, University of Colorado Denver, 12800 E. 19th Avenue, Aurora, CO 80045, USA; 3 Center for Personalized Diagnostics, Arizona State University, 1001 S. McAllister Avenue, Tempe, AZ 85287-6401, USA

## Abstract

**Introduction:**

Juvenile idiopathic arthritis (JIA) is a heterogeneous disease characterized by chronic joint inflammation of unknown cause in children. JIA is an autoimmune disease and small numbers of autoantibodies have been reported in JIA patients. The identification of antibody markers could improve the existing clinical management of patients.

**Methods:**

A pilot study was performed on the application of a high-throughput platform, the nucleic acid programmable protein array (NAPPA), to assess the levels of antibodies present in the systemic circulation and synovial joint of a small cohort of juvenile arthritis patients. Plasma and synovial fluid from 10 JIA patients was screened for antibodies against 768 proteins on NAPPAs.

**Results:**

Quantitative reproducibility of NAPPAs was demonstrated with > 0.95 intra-array and inter-array correlations. A strong correlation was also observed for the levels of antibodies between plasma and synovial fluid across the study cohort (*r = *0.96). Differences in the levels of 18 antibodies were revealed between sample types across all patients. Patients were segregated into two clinical subtypes with distinct antibody signatures by unsupervised hierarchical cluster analysis.

**Conclusion:**

The NAPPAs provide a high-throughput quantitatively reproducible platform to screen for disease-specific autoantibodies at the proteome level on a microscope slide. The strong correlation between the circulating antibody levels and those of the inflamed joint represents a novel finding and provides confidence to use plasma for discovery of autoantibodies in JIA, thus circumventing the challenges associated with joint aspiration. We expect that autoantibody profiling of JIA patients on NAPPAs could yield antibody markers that can act as criteria to stratify patients, predict outcomes and understand disease etiology at the molecular level.

## Introduction

Juvenile idiopathic arthritis (JIA) is an autoimmune disease and the second most common disease of childhood after diabetes, affecting approximately one in every 1,000 children [1]. JIA consists of a clinically heterogeneous group of arthritic disorders that begin before age 16 years and persist for more than 6 weeks. The cause and pathogenesis of JIA are still poorly understood. Chronic inflammation of joints is the only common denominator for all JIA patients. Of the seven subsets of JIA identified according to the International League Against Rheumatism classification, oligoarticular, extended oligoarticular and polyarticular are the most common [2]. Adverse outcomes can present to varying degrees regardless of the disease subtype [3]. In approximately 25% of children with oligoarticular JIA the disease spreads over time to involve many joints, a condition known as extended oligoarticular disease [4]. A number of major challenges exist in the management of JIA, including a clear and timely diagnosis of JIA, identification of patients at risk of an aggressive disease course, and prediction of response to standard treatment. In overcoming these challenges the central aim is to prevent joint and periarticular damage.

Although clinical manifestations imply the involvement of adaptive immunity in JIA, research into the range of autoantigens that drive the humoral response has significantly lagged behind that of genetic analyses. Genetic associations between JIA subtypes and HLA or non-HLA molecules are known to be related to immune response and associated with other autoimmune diseases [5,6]. Autoimmune phenomena such as autoreactive T cells and autoantibodies can be detected readily in most JIA subgroups [7-9]. In contrast to the majority of circulating proteins, autoantibodies are easy to measure, less subject to variation in the blood, remarkably stable in serum samples, and readily detected with well-validated secondary reagents. Autoantibody tests for anti-cyclic citrullinated antibody, rheumatoid factor (RF) and antinuclear antigen (ANA) status have been adopted into clinical practice, and their role in diagnosis is well established in adult autoimmune diseases [10].

Autoantibody study in JIA has generally followed in the wake of research into adult rheumatoid arthritis (RA) by testing the performance of RA autoantigen targets in JIA patients. Even though the evidence provided by this approach has been limited, these studies have provided a glimpse of the heterogeneous molecular mechanisms that contribute to the pathogenesis of various subtypes of JIA. The heterogeneity of JIA is exemplified by the fact that findings from RA can only be applied to a few specific subgroups of JIA patients. RF, which has a sensitivity of ~80% in RA, is present only in a small subgroup of patients with polyarticular-onset JIA [11]. The discovery of RA-specific autoantibodies against citrullinated proteins prompted tremendous interest in their performance in JIA patients. Autoantibodies against citrullinated proteins (anti-cyclic citrullinated protein), which are highly specific for RA, can only be detected in RF-positive JIA patients [12,13], but are associated with a higher risk of joint erosion, more aggressive disease and therapeutic resistance [14,15]. Circulating immune complexes containing citrullinated fibrinogen have been identified in only a small subset of JIA patients [16]. Antinuclear antibodies, in contrast, are present in a significant proportion of children with oligoarticular, extended oligoarticular and polyarticular disease, and their presence, to some extent, predicts the development of uveitis.

Although reliable and sensitive, the ELISA is not practical as a high-throughput platform for screening large numbers of autoantibodies. Protein microarrays, on the other hand, provide a multiplexed, high-throughput platform to profile thousands of antibodies in parallel. Despite the potential of harnessing information on the humoral immune response in JIA, protein microarrays have only recently been used to investigate JIA [17]. The current study applies an innovative protein array platform, the nucleic acid programmable protein array (NAPPA), to provide initial evidence of the true variety of autoantibodies present in JIA patients. The NAPPA overcomes some challenges associated with conventional high-density protein arrays in terms of cost and programmability, and makes it possible to screen hundreds of samples for the discovery phase and to design customized arrays for the validation phase [18,19].

As an exploratory study into the autoantibody complement of early-stage juvenile arthritis, the goals were to determine whether NAPPAs are reproducible enough for autoantibody screening of JIA patients, whether plasma can be used in place of synovial fluid to reflect autoantibodies pertinent to joint pathology, and whether distinct autoantibody expression patterns can be observed for specific clinical subtypes. Both plasma and joint (synovial) fluid were therefore analyzed, and results were compared between duplicate spots within individual arrays, within the same sample type across different patients, and between sample types to highlight potential correlations and differences in autoantibody levels. This strategy highlights the performance characteristics and exhibits the inter-sample and intra-sample comparison abilities of the NAPPA platform. Associations with existing clinical decision-making criteria such as C-reactive protein (CRP) and functional ontology of autoantigens with differentially expressed autoantibodies were also explored.

## Materials and methods

### Patient samples

Ten patients with newly diagnosed untreated JIA according to International League Against Rheumatism criteria entered this study and were followed for 1 year. At the time of initial sampling there were six children with oligoarticular arthritis and four with polyarticular arthritis (three of whom were RF-negative). At 1 year, three oligoarticular cases had been reclassified as having extended oligoarticular JIA. All patients were examined by a consultant rheumatologist (MER), who confirmed their diagnosis. For the purposes of this study, only initial synovial fluids from children with disease duration < 6 months and untreated disease were studied. Arthrocentesis and joint steroid injection were performed according to clinical need.

Clinical details recorded included the subtype of JIA, age, sex, erythrocyte sedimentation rate (ESR) and CRP. Local inflammation was defined both as joint swelling and/or pain and tenderness with a reduced range of movement on physical examination. All synovial fluids were aspirated using an aseptic technique; plasma was obtained at the same visit. Samples were immediately centrifuged (5,000×*g*, 15 minutes, 4°C), aliquoted and stored (at -80°C) for at least 1 year to allow for clinical reclassification. Medical Ethics Committee approval was obtained for this study at Green Park Healthcare Trust, and patient assent and parent informed consent were given (Office for Research Ethics Committees Northern Ireland 408/03).

### Nucleic acid programmable protein array production

A total of 768 sequence-verified full-length human genes in pANT7_cGST or pLDNT7_nFLAG were obtained from the Center for Personalized Diagnostics at the Arizona State University and are publicly available [20]. The 768 genes were selected to reflect a broad variety of physiological and pathological pathways (Tables S1 and S2 in Additional File 1). The high-throughput preparation of high-quality supercoiled DNA for cell-free protein expression was performed as described [19]. Briefly, *Escherichia-coli*-bearing expression plasmids were grown in 1.5 ml terrific broth for 24 hours with appropriate antibiotics. Plasmid DNA was purified with the NucleoPrep II anion exchange resin (Macherey-Nagel Inc., Bethlehem, PA, USA) using a Biomek FX (Beckman Coulter, Inc., Fullerton, CA, USA) automated laboratory workstation. Automated addition of all solutions was accomplished using a Matrix WellMate (Thermo Scientific, Hudson, NH, USA) rapid bulk liquid-dispensing instrument. Purified DNA was precipitated by addition of 0.6 volumes of isopropanol, followed by centrifugation at 5,000 ×*g *for 30 minutes. The DNA pellet was washed with 200 μl of 80% ethanol, centrifuged at 5,000 ×g for 15 minutes, dried and resuspended in deuterated water.

Plasmid DNA was supplemented with capture antibody (50 μg/ml anti-glutathione *S*-transferase (anti-GST) antibody (GE Healthcare Biosciences, Piscataway, NJ, USA) or anti-FLAG antibody (Sigma-Aldrich, St Louis, MO, USA)), protein cross-linker (2 mM BS3; Pierce, Rockford, IL, USA) and BSA (3 mg/ml; Sigma-Aldrich) prior to printing onto the array surface. All samples were printed using a Genetix QArray2 with 300 μm solid tungsten pins on aminosilane-coated glass slides. Arrays were stored in an airtight container at room temperature until use. The printed DNA was transcribed and translated *in situ *using previously published protocols [19]. The quality of DNA printing was assessed by PicoGreen staining (Invitrogen, Carlsbad, CA, USA) and protein expression using anti-GST mAb (Cell Signaling, Danvers, MA, USA) diluted at 1:200.

### Detection of sample antibodies

For autoantibody profiling, printed arrays were after expression incubated with plasma or synovial fluid diluted 1:600 in the dilution buffer (5% milk with 0.2% Tween 20 in PBS) at 4°C overnight with gentle mixing. Human antibodies reacting with expressed proteins on array were detected by Cy5-labeled anti-human IgG (Jackson ImmunoResearch Laboratories, West Grove, PA, USA). Slides were scanned with a Tecan powerscanner and the images were quantified using ArrayPro Image analysis software. The highly immunogenic Epstein-Barr virus-derived antigen (Epstein-Barr virus nuclear antigen 1) was included as the positive control antigen. Negative controls included empty vectors and no DNA controls. Registration spots for array alignment were printed purified human IgG proteins.

### Differential expression and unsupervised cluster analysis

Ten plasma samples and 10 matched synovial fluid samples from JIA patients were screened on 768 antigens displayed in NAPPA format. The normalized volume for each spot on each array was calculated by first removing the background signal estimated by the first quartile of the nonspots, and then log-transformation of the spot volumes was used to generate normally distributed data. The log-normalized volume was used to compare spot abundance. Differential spot analysis was performed across the two sample types with Prism 5.03 software (GraphPad Software Inc., La Jolla, CA, USA). Each comparison was filtered to find spots having > 1.45-fold change in average normalized volume expression between the sample types.

Expression data were analyzed using Epclust, a generic data clustering, visualization and analysis tool [21]. Unsupervised hierarchical analysis reordered protein expression patterns in an agglomerative fashion, using the unweighted pair-group average with arithmetic mean clustering procedure. Euclidean ranked correlation was the similarity measure used to group or separate the expression data. A heatmap was produced, accompanied by dual dendrograms depicting the extent of similarity between different patients and autoantibody levels in the samples. Information on the functional ontology of target antigens was mined from the UniProt archive version 2011.03.11 [22].

### Serine/threonine kinase-10 antibody ELISA

As the NAPPA is a novel platform and is not routinely used for clinical measurements, independent verification of patient antibody levels to a protein randomly selected from Table 1 was performed by ELISA. GST protein fused to the full-length serine/threonine kinase-10 gene was expressed using the rabbit reticulocyte lysates, as above. Serine/threonine kinase-10-GST protein was applied to an anti-GST-coated ELISA plate (GE Biosciences, Pittsburgh, PA, USA) overnight at 4°C. Plates were washed in PBS-0.05% Tween and blocked with PBS-Tween with 2% milk, overnight at 4°C. After washing, the plate was incubated with 1:300 diluted plasma or synovial fluid samples and the presence of autoantibodies against serine/threonine kinase-10 was detected by incubation with horseradish peroxidase-labeled anti-human IgG secondary antibody (Zymax; Invitrogen). Tetramethyl benzidine substrate (Dako, Carpinteria, CA, USA) was then added and the reaction stopped with 1 M H_2_SO_4_. The 450 nm optical density signals were read on a SpectralMax plate reader (Molecular Devices Inc., Sunnyvale, CA, USA). Protein ELISA analyses were performed in duplicate for each of the 10 paired patient fluids. Mean absorbance readings were subjected to paired *t *test analyses to determine the significance of any variation in antibody levels between fluids.

**Table 1:** Autoantibodies differentially expressed between circulation and joint

Expression	Autoantigen	UniProt accession	Gene name	Functional ontology biological process
Higher in PL (> 45%)	C-terminal binding protein 1	Q13363	CTBP1	Differentiation, host-virus interaction, transcription regulation
	Guanine nucleotide binding protein (G protein), alpha 13	Q14344	GNA13	Cellular component movement, platelet activation
	Presenilin associated, rhomboid-like protein	Q9H300	PARL	-
	Trans-2,3-enoyl-CoA reductase	Q9NZ01	GPSN2	Fatty acid biosynthesis, lipid synthesis
	Cas-Br-M (murine) ecotropic retroviral transforming sequence b	Q13191	CBLB	Ubiquitin conjugation pathway
	Abhydrolase domain containing 16A	O95870	BAT5	-
	Tubulin, gamma complex associated protein 3	Q96CW5	TUBGCP3	Microtubule nucleation at the centrosome
	Modulator of apoptosis 1	Q96BY2	MOAP1	Apotosis
	Family with sequence similarity 76, member B	Q5HYJ3	FAM76B	-
	Thyroid hormone receptor, alpha	P10827	THRA	Nuclear hormone receptor, transcriptional regulation
	Apolipoprotein A-II	P02652	APOA2	Host-virus interaction, lipid transport
	Eyes absent homolog 2 (Drosophila)	O00167	EYA2	DNA damage and repair, transcription regulation
	IL-6 receptor	P08887	IL6R	Regulation of the immune response, acute-phase reactions and hematopoiesis
	G protein-coupled receptor 153	Q6NV75	GPR153	-
	Histone deacetylase 3	O15379	HDAC3	Anti-apoptosis, histone deacetylation, transcriptional regulation
	Actin related protein M1	Q9BYD9	ARPM1	-
	Natural killer cell group 7 sequence	Q16617	NKG7	-
	Mitogen-activated protein kinase kinase 7	O14733	MAP2K7	Activation of JUN kinases
	Ubiquitin-conjugating enzyme E2A (RAD6 homolog)	P49459	UBE2A	DNA damage and repair, ubiquitin conjugation pathway
	v-kit Hardy-Zuckerman 4 feline sarcoma viral oncogene homolog	P10721	KIT	-
	Pleckstrin homology domain containing, family O member 1	Q53GL0	PLEKHO1	Regulation of the actin cytoskeleton
	Adaptor-related protein complex 1, sigma 1 subunit	P61966	AP1S1	Endocytosis, protein transport
	neuregulin 1	Q02297	NRG1	Induces growth and differentiation of epithelial, glial, neuronal and skeletal muscle cells
	Peroxisomal biogenesis factor 5	P50542	PEX5	Protein transport
	Nuclear receptor subfamily 6, group A, member 1	Q15406	NR6A1	Transcriptional regulation, cell proliferation
	Serine/threonine kinase 10	O94804	STK10	Serine and threonine phosphorylation
	Eukaryotic translation initiation factor 1	P41567	EIF1	Protein biosynthesis through ribosomal complexes
	Zinc finger protein 36, C3H type-like 2	P47974	ZFP36L2	Cell proliferation, regulates response to growth factors
	Cartilage oligomeric matrix protein	P49747	COMP	Controls apoptosis and cell adhesion within extracellular matrix
Higher in SF (> 45%)	Actinin, alpha 1	A1L0V1	ACTN1	-
Similar levels in PL and SF (< 10%)	Protein phosphatase 2, catalytic subunit, beta isozyme	P62714	PPP2CB	Protein dephosphorylation
	Peroxisomal biogenesis factor 10	O60683	PEX10	Peroxisome biogenesis
	mitogen-activated protein kinase kinase kinase 7	O43318	MAP3K7	Component of a protein kinase signal transduction cascade; Stimulates NF-κB activation and the p38 mitogen-activated protein kinase pathway
	3-Hydroxy-3-methylglutaryl-CoA synthase 1	Q01581	HMGCS1	Cholesterol, steroin and sterol biosynthesis; lipid synthesis
	TNF receptor-associated protein 1	Q12931	TRAP1	Chaperone that expresses an ATPase activity
	Troponin C type 1 (slow)	P63316	TNNC1	Regulatory protein of striated muscle contraction

Target antigen list of a select group of autoantibodies that were detected either at similar levels or with at least a 45% difference between synovial fluid (SF) and plasma (PL). The majority of the differentially expressed antibodies are raised in plasma; for example, apolipoprotein AII and cartilage oligomeric matrix protein (COMP) [Uniprot:P02652, Uniprot:P49747]. Only actinin alpha I targeted antibodies were detected at higher levels in SF within the 45% difference threshold [Uniprot:A1L0V1]. Six proteins to which mean antibody levels remained static between fluids have also been listed for comparison. The levels of antibodies against these proteins across individual patient samples are shown in Figure 2A and Figure 3C. STK10, serine/threonine kinase-10.

## Results

### Patients

As measures of the acute-phase reaction, the plasma ESR and CRP reflect the status of patients when samples were collected (Table 2). When patients are ranked from those displaying the lowest levels of inflammation to the highest, two main groups could be defined: low-medium inflammation (Patients O3, E2, P3, O1, E1 and P1); and medium-high inflammation (Patients O2, P4, E3 and P2). With particular relevance to the study aim, one-half of the study patients were positive for routinely measured autoantibodies. RF was detected in only one patient (Patient P2), whereas ANA antibodies were present in four patients (Patients O1, O2, E1 and P4).

**Table 2:** Patient demographics

Patient	JIA subgroup (at 1 year)	Sex	Age at sample (years)	WBC (×10^6 ^cells/l)	ESR (mm/hour)	CRP (mg/l)	RF	ANA titer
O1	Oligoarticular	Male	6.6	8.2	10.0	15.0	Negative	1:80
O2	Oligoarticular	Female	2.5	12.8	37.0	26.0	Negative	1:80
O3	Oligoarticular	Male	8.5	6.6	2.0	6.4	Negative	Negative
E1	Extended Oligoarticular	Female	7.5	9.5	22.0	9.0	Negative	1:80
E2	Extended Oligoarticular	Female	4.7	10.9	10.0	8.4	Negative	Negative
E3	Extended Oligoarticular	Female	11.0	9.1	62.0	66.5	Negative	Negative
P1	Polyarticular	Female	3.2	11.0	31.0	4.0	Negative	Negative
P2	Polyarticular	Female	16.6	11.8	110.0	72.0	231.0	Negative
P3	Polyarticular	Female	2.2	7.5	10.0	8.6	Negative	Negative
P4	Polyarticular	Female	2.6	10.1	45.0	19.1	Negative	1:320

Demographic characteristics and routine laboratory measures of the study subjects. ANA, antinuclear antigen; CRP, C-reactive protein; ESR, erythrocyte sedimentation rate; JIA, juvenile idiopathic arthritis; RF, rheumatoid factor; WBC, white blood cells.

### Intra-slide precision and inter-sample correlation

**Figure 1:**
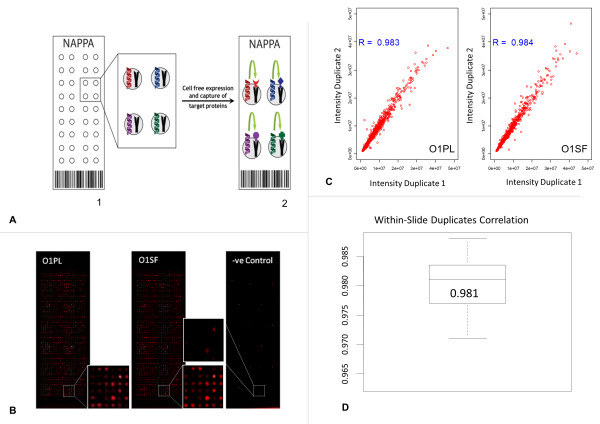
**Intra-slide duplicate spot correlation**. **(A1) **Nucleic acid programmable protein array (NAPPA) spotted with genes of interest. **(A2) **All proteins are tagged at the C-terminus to ensure only full-length translated proteins can be captured *in situ *by cospotted anti-tag antibodies. NAPPA has consistent protein amounts displayed at each spot; most are within twofold of the average [18,19]. Proteins are expressed just in time for assay, which eliminates concern of protein stability. **(B) **The 768 antigen NAPPAs incubated with plasma (O1PL), synovial fluid (O1SF) and PBS (to control for detection antibody specificity). Arrays were rinsed and incubated with a fluorophore conjugated anti-human IgG detection antibody, to probe for antigen-bound antibodies originating from the sample. **(C) **Scatter plots of data that illustrate the correlation between duplicate protein antigen spots within the same slide. Correlation of all 768 antigen duplicates from a single patient for plasma (O1PL; *r *= 0.983) and synovial fluid (O1SF; *r *= 0.984) incubated slides. **(D) **Correlation values of intra-slide duplicate values for each patient sample (plasma and synovial fluid, *n *= 20) plotted to illustrate the precision of measurements across the experiment. An average correlation coefficient of *r *= 0.981 is observed across all samples (range of *r *= 0.971 to 0.988). **{Figure 1D needed to be redrawn; please find new image for whole figure}**

In total, 768 antigen NAPPAs were probed with plasma, synovial fluid, and the dilution buffer (to control for detection antibody specificity) (Figure 1A and 1B). Scatter plots of data that illustrate the correlation between duplicate protein antigen spots within the same incubated slide from a single patient for plasma (Patient O1, *r *= 0.98) and synovial fluid (Patient O1, *r *= 0.98) are shown in Figure 1C. Correlation values of intra-slide duplicate values for each patient sample (plasma and synovial fluid, *n *= 20) were plotted to illustrate the precision of measurements across the experiment. An average correlation coefficient of *r *= 0.98 is observed across all samples (range of *r *= 0.97 to 0.99) (Figure 1D).

**Figure 2:**
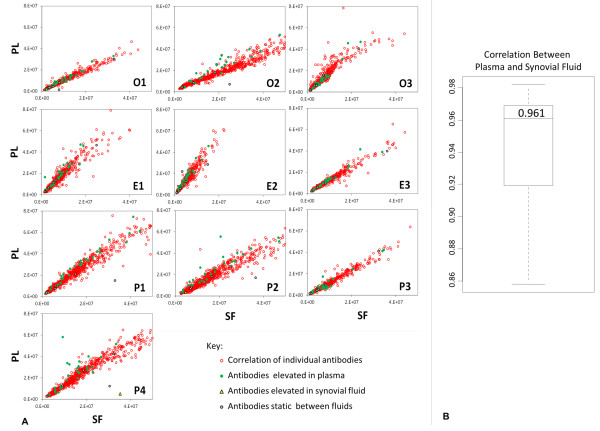
**Inter-slide correlation between sample types**. **(A) **Scatter plot correlation of antibody-antigen spot signal intensity between plasma (PL) and synovial fluid (SF) samples on a patient-by-patient basis. Across the study cohort, antibody prevalence in plasma is evident from the increased signal scale. Antibodies detected at ≤45% higher levels within plasma are indicated by green circular markers; those detected at ≤45% higher levels within synovial fluid are indicated by yellow triangular markers; a representative set detected at similar mean levels in both sample types are indicated by black circular markers. This subset of antibody targets is summarized in Table 1. **(B) **Overall mean correlation between fluids across all antibodies detected within all samples is *r *= 0.961 (range of *r *= 0.859 to 0.981).

A strong correlation of antibody-antigen spot signals exists between synovial fluid and plasma samples on a patient-by-patient basis. A wider range exists in inter-slide correlations, with an overall trend toward antibody predominance in plasma. Outliers evident within correlations indicate a select group of antibodies that are detected at higher levels in one sample type within specific individuals (that is, Patients O1, O2, P1, P3 and E3; Figure 2A). The overall mean correlation between fluids across all antibodies detected within all samples is *r *= 0.96 (range of *r *= 0.86 to 0.98; Figure 2B).

### Differential expression of antibodies between the circulation and joint

**Figure 3:**
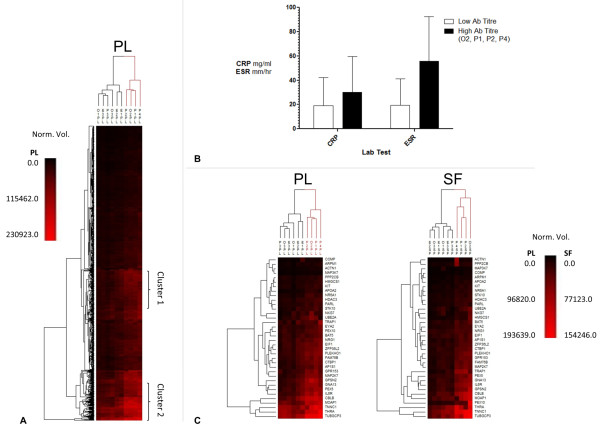
**Heatmap display of antibody expression pattern**. **(A) **Hiearchical cluster analysis of all 768 screened antibodies ordered by Euclidean distance measure (unweighted pair-group average with arithmetic mean) for plasma (PL) from all 10 patients, revealing distinguishing expression patterns. The degree of antibody binding is reflected in the color intensity of the heatmap, whereby the more intense the red, the more antibody is bound to a given antigen. Clustering and seriation of both antibody target ID's in rows and individual patient samples in columns segregates the study cohort by antibody expression levels. Two core patient clusters are revealed: lower antibody levels (Patients O1, O3, E1, E2, E3 and P3); and higher antibody levels (Patients O2, P1, P2 and P4) (red in patient sample dendrogram). **(B) **Levels of the conventional laboratory measures C-reactive protein (CRP) and erythrocyte sedimentation rate (ESR) are plotted for the study cohort with patients split into two groups defined above in (A) by low or high antibody levels. Error bars represent standard deviation; no significant difference exists between the two groups. **(C) **Hierarchical cluster analysis of autoantibody levels detected with differences of at least 45% between synovial fluid (SF) and PL. The majority of these antibodies are elevated in plasma relative to synovial fluid.

Unsupervised hierarchical cluster analysis of reactivity data against all 768 screened antigens based on the Euclidean distance measure (unweighted pair-group average with arithmetic mean) for both plasma and synovial fluid from all 10 patients revealed distinguishing expression patterns (Figure 3A). Constructed dendrograms for both antibody targets and individual patient samples indicate how the expression levels of proteins that are subject to similar regulatory pathways cluster and how these may relate to clinical subgroups within the study cohort. Tables S3 and S4 in Additional file 1 and Figure S1 in Additional file 2 list the proteins involved in regulating two clusters highlighted in Figure 3A. Cluster 1 proteins are more prevalent in Patients O2, P1, P3 and P4, whereas cluster 2 is elevated across the whole patient cohort. A given transcriptional regulator can impact upon multiple target genes - but it is interesting to note that even though each cluster contains the same number of proteins (*n *= 49), cluster 1 is subject to control by a smaller number (*n *= 42) of transcriptional regulators than those in cluster 2 (*n *= 95).

Patient samples were clustered into two key groupings: low antibody levels (Patients O1, O3, E1, E2, E3 and P3); and high antibody levels (Patients O2, P1, P2 and P4). It is intriguing that three of the four at-risk patients previously identified with medium to high levels of CRP or ESR (Patients O2, P2 and P4) also cluster independently with high levels of antibodies relative to the remaining patients (Figure 3B). The trends observed do not reach statistical significance by Student's *t *test.

**Figure 4:**
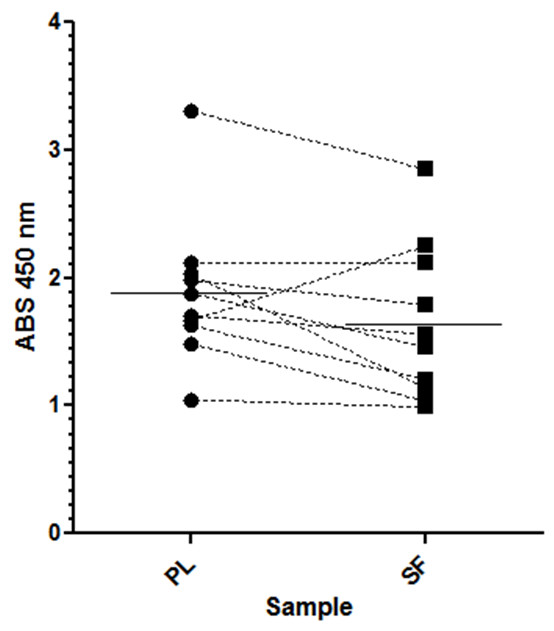
**ELISA verification of serine/threonine kinase-10 antibody levels**. The plasma concentrations of anti-serine/threonine kinase-10 antibodies were confirmed to be significantly higher in plasma (PL) than in synovial fluid (SL) by ELISA (paired *t *test *P *= 0.038). The paired PL (circular marker) and SF (square marker) samples from individual juvenile idiopathic arthritis patients are identified by a dashed line between points. The mean ELISA reading at 450 nm across the study cohort for each sample type is represented by a horizontal solid line.

Unsupervised hierarchical cluster analysis of autoantibody levels detected with differences of at least 45% between synovial fluid and plasma is shown in Figure 3C. This difference threshold produced a workable list of 36 antibodies targeting specific antigens (Table 1). The majority of the differentially expressed antibody levels are elevated in plasma - that is, apolipoprotein AII, IL-6 receptor, thyroid hormone receptor alpha and G-protein coupled receptor 153 [Uniprot:P02652, Uniprot:P08887, Uniprot:P10827, Uniprot:Q6NV75]. Only actinin alpha I targeted antibodies were detected at higher levels in synovial fluid within the 45% difference threshold [Uniprot:A1L0V1]. The modest although significant increase in serine/threonine kinase-10 antibodies was independently confirmed by ELISA (*P *= 0.038; Figure 4) [Uniprot:O94804].

## Discussion

The production of antibodies against self-antigens (autoantibodies) is a characteristic feature of many autoimmune diseases. At a clinical level, tests for specific autoantibodies - such as ANA positivity - are routinely employed to aid the diagnosis and to track the progress of these diseases. Traditionally, autoantibodies have been identified with a one-antigen-at-a-time, hypothesis-driven approach using methods such as immunofluorescence and ELISA.

In contrast, microarrays provide a particularly effective platform for the systematic study of thousands of proteins in parallel because they are sensitive and require low sample volumes [23,24]. Protein microarrays involve the display of thousands of different proteins with high spatial density on a microscopic surface. Protein microarrays have been applied to autoimmune biomarker studies focused on presymptomatic screening and diagnosis, clinical outcome prognosis and therapeutic response prediction [25-28]. In the field of autoimmune disorders, conventional printed arrays have been used to study RA, systemic lupus erythematosus, multiple sclerosis, hepatitis and encephalomyelitis [29-34].

The NAPPA is an innovative method to produce protein microarrays, where cDNAs encoding proteins of interest are spotted onto activated surfaces and proteins are produced *in situ *using mammalian *in vitro *expression systems [18,19]. The freshly made protein is captured by cospotted antibodies specific for a tag encoded at the end of the amino acid sequence. This approach circumvents the labor and cost considerations associated with conventional spotting of labile recombinant proteins into arrays. NAPPA technology recently revealed that ankylosing spondylitis patients' autoantibody responses were targeted towards connective, skeletal and muscular tissue, unlike those of RA patients [35].

The development of protein microarrays offers a compelling strategy to comprehensively screen antibodies. When properly executed, these microarrays allow thousands of full-length proteins to be tested simultaneously. NAPPA technology replaces printing proteins with a more reliable and less expensive process of printing DNA, circumventing the need to express, purify and store proteins. Additionally, this avoids concerns about protein stability because the proteins are expressed only at the time of assay. The responses are rapidly identified because the address of each protein is known in advance and there are no representation issues; all proteins, even rare ones, are represented equally (usually in duplicate). The proteins are arrayed on a single microscope slide requiring only a few microliters of plasma or serum per assay. An additional advantage of this approach is that the data can be evaluated to look for both informative individual antigens as well as for patterns of antigen responses with good predictive value [17,36,37]. Moreover, the coupling of clustering algorithms with the simultaneous comparison of many antigen responses lends itself well to the determination of antigens with concordant and independent responses.

In the current study, associations were found between at-risk patients with elevated CRP or ESR and elevated antibody levels. The trend is observed in both plasma and synovial fluid; and although no statistical significance can be tied to this association, future study with an expanded cohort could clarify any relationship. Given the relatively low prevalence of individual autoantibodies, large numbers of patient and control sera are needed to demonstrate statistical confidence in new markers. There is thus a strong need for quantitative reproducibility both within the array and from array to array so that data can be analyzed across arrays probed with samples on different days to draw statistical conclusions.

The current investigation clearly demonstrated quantitative reproducibility, differential reactivity against different proteins for individual samples, differential reactivity among different patients, and the potential to use plasma samples as a source for multiplex autoantibody discovery in JIA. The strong correlation between synovial fluid and plasma antibody levels represents a novel finding and provides confidence to use plasma for discovery of autoantibodies also found in the pathological joint, thus circumventing the challenges associated with joint aspiration. Certain antibodies were identified at elevated levels in plasma (relative to synovial fluid), suggesting that these may emanate either from immune cells within the circulation or from other tissues around which blood circulates. Additionally, these elevated antibodies were possibly produced just prior to sample collection, in response to stimulatory antigens, and have not yet reached homeostatic levels between the circulation and joint fluids. Intriguingly, in some cases there is a shift toward a higher signal in synovial fluid; however, this did not alter the relative abundances of individual species. One possible explanation of this variation in antibody concentration is the hydration status of the subjects in question. Overall, however, the relative abundance of specific antibody species correlates strongly in most cases.

A wealth of work exists based on conventional tests for solitary autoantibody species in plasma or serum across a number of arthritides, including JIA. ANAs are mainly associated with oligoarticular-onset JIA patients, but are also present in polyarticular and psoriatic subtypes [38,39]. Several reports have documented the association between the presence of ANAs and uveitis in JIA patients [40]. Distinct autoantigens that contribute to ANA positivity have not yet been identified. ANA testing is still performed by indirect immunofluorescence on HEp2 cells [41]. Antibodies against histones, HNRPA2 and nonhistone chromosomal proteins have been reported in JIA patients [42]. However, ANAs are present in patients with other autoimmune diseases as well as healthy individuals, and might be transiently increased during viral or bacterial infections. ANA testing to diagnose JIA is therefore limited due to its low disease specificity. Several other autoantigens, such as heat shock glomerular and endothelial proteins, have also been assessed in JIA, but the results are inconsistent and of limited value either for diagnosis or for understanding the disease pathological mechanism [43-45].

The unsupervised pair-wise clustering of antibody levels and clinical subtype, albeit in a small cohort, produced some interesting observations. The expression levels of antibodies directed at a select group of antigens appear to segregate patients into two clinically relevant clusters. The two defined patient groups are distinguished by the severity of disease, whereby a higher antibody titer is associated with an increase of the acute-phase indicators ESR and CRP. Additionally, a number of these high-antibody-titer patients tested ANA-positive or RF-positive, suggesting a trend - although this would require confirmation in a much larger patient cohort. Similarly, a recent protein array study demonstrated that juvenile arthritis patients can be stratified into distinct molecular subtypes based on the expression of antibody signatures [17]. Stoll and colleagues demonstrated that autoantibodies directed at the extracellular matrix and nuclear antigens are elevated in a subgroup of oligoarticular JIA patients that were less likely to attain clinical remission. Likewise, clinical subtyping of adult RA patients by arrays of 10 preselected antigens has been successfully demonstrated on an Immunological Multi-Parameter Chip Technology platform produced by Roche Diagnostics Corp., Indianapolis, IN, USA [46]. As a constituent of the observed elevated signature, antibodies against the IL-6 receptor, for example, may signal a distortion in the regulation of the immune and acute phase responses. In fact, synthetic humanized anti-IL-6 receptor embodies one of the highly selective biologic therapeutic tools now available to clinicians [47].

## Conclusion

In the future we intend to move this work towards a statistically powered discovery study that will analyze a much larger cohort with healthy control subjects, to capture significant biological variance in antibody levels that correlate with clinically relevant outcomes. Pertinent clinical questions focused on the management of arthritis patients that remain unanswered include the prognosis of adverse outcome (for example, spread of inflammation to previously unaffected joints) and the timely prediction of which drugs could abrogate that outcome (for example, heterogeneity in response amongst JIA subgroups to expensive biologic drugs). Discovery findings should be considered a set of leads that then require meticulous validation, especially with respect to the utility of a multiplex antibody signature in a routine clinical setting.

In summary, autoantibody research in JIA has in general been hypothesis driven and often adapted from findings of adult RA and other autoimmune diseases. Unfortunately, even the most established findings in RA have not proven useful and relevant in all subtypes of JIA. A comprehensive profiling of the autoantigen repertoire in JIA patients will greatly improve our understanding of the molecular and cellular mechanisms that lead to the loss of immunological tolerance and production of autoantibodies.

## Abbreviations

ANA: antinuclear antigen; BSA: bovine serum albumin; CRP: C-reactive protein; ELISA: enzyme-linked immunosorbent assay; ESR: erythrocyte sedimentation rate; GST: glutathione-*S *transferase; IL: interleukin; JIA: juvenile idiopathic arthritis; mAb: monoclonal antibody; NAPPA: nucleic acid programmable protein array; PBS: phosphate-buffered saline; RA: rheumatoid arthritis; RF: rheumatoid factor.

## Competing interests

The authors declare that they have no competing interests.

## Authors' contributions

DSG designed the study, drafted the manuscript and performed statistical analysis. JQ helped conceive the study, participated in the image analysis of NAPPA slides and helped draft the manuscript. EAM participated in the printing and protein translation of NAPPA slides. KB carried out the immunoassays. MER collated the patient samples and demographic data. JL conceived the study and participated in its coordination and helped draft the manuscript. All authors read and approved the final manuscript.

## Supplementary Material

Additional file 1 **Table S1, a complete list of NAPPA proteins, presenting the Entrez gene symbol, protein name, cellular location and molecule type for each protein produced on the NAPPA array within the current study; Table S2, functional networks of NAPPA proteins, listing the physiological and pathological functions of 25 networks constructed from the constituents of the NAPPA arrays used within the current study; Table S3, cluster 1 transcriptional regulation, listing the proteins responsible for transcriptional regulation of the target antigens within cluster 1 (**Figure 3A**and **Additional File 2 **); and Table S4, cluster 2 transcriptional regulation, listing the proteins responsible for transcriptional regulation of the target antigens within cluster 2 (**Figure 3A**and **Additional File 2**)**. Click here for file

Additional file 2 **Figure S1, transcriptional regulation of antigen clusters, showing magnified sections of the hierarchical cluster analysis heatmap in **Figure 3A**to illustrate two distinct clusters of 49 proteins targeted by antibodies within the plasma of study subjects**. The proteins responsible for transcriptional regulation of the target antigens within cluster 1 and cluster 2 are listed in Tables S3 and Table S4 in Additional File 1, respectively. Click here for file
