# Supplementary figures and images for: Circulating and synovial antibody profiling of juvenile arthritis patients by nucleic acid programmable protein arrays
Source: Arthritis Res Ther. 2012 Apr 17;14(2):R77. doi: 10.1186/ar3800 (PMC3446451; doi:10.1186/ar3800)

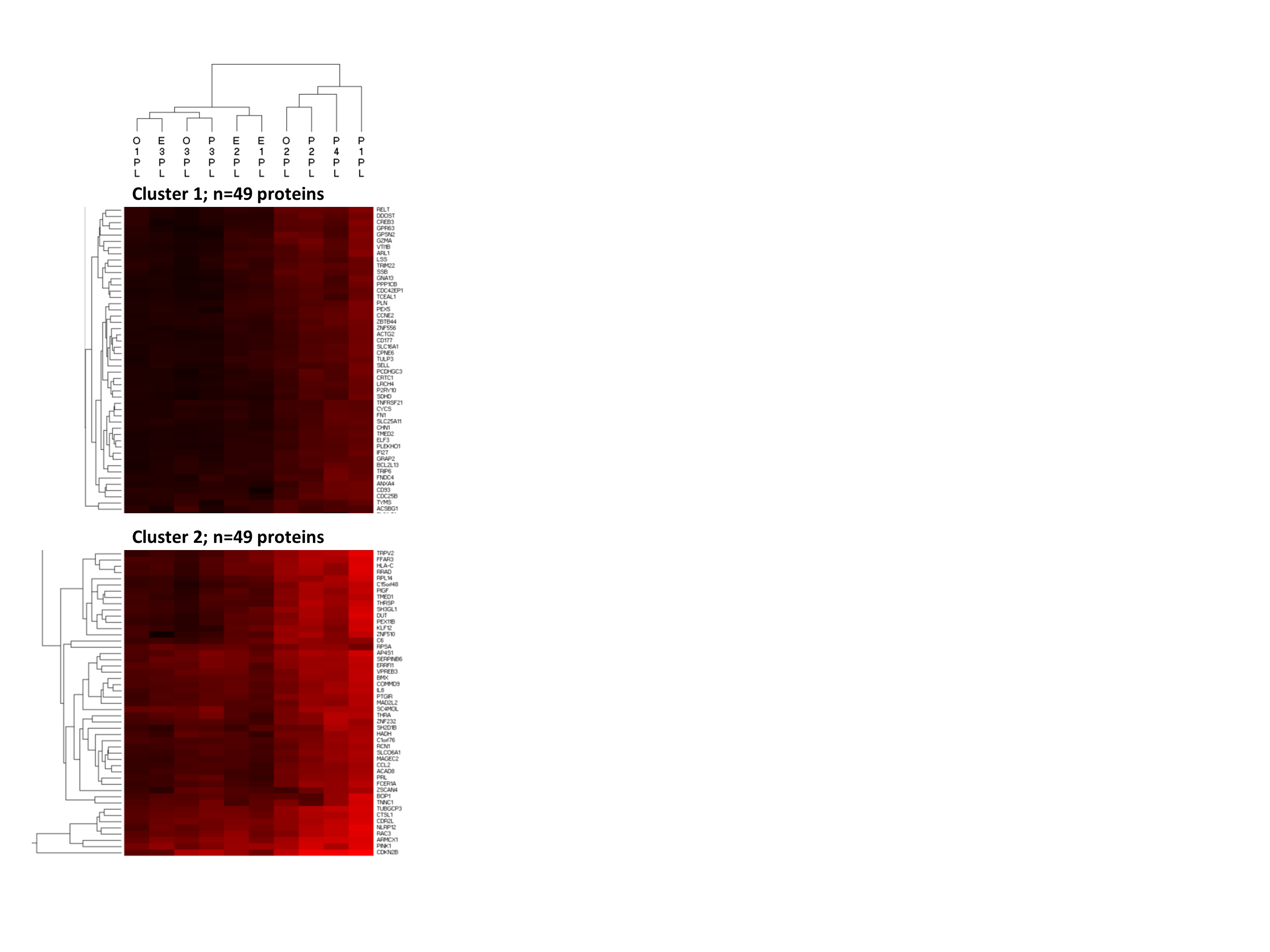

Supplement: Additional file 2 — Figure S1, transcriptional regulation of antigen clusters, showing magnified sections of the hierarchical cluster analysis heatmap in Figure 3Ato illustrate two distinct clusters of 49 proteins targeted by antibodies within the plasma of study subjects. The proteins responsible for transcriptional regulation of the target antigens within cluster 1 and cluster 2 are listed in Tables S3 and Table S4 in Additional File 1, respectively. [file ar3800-S2.TIFF]
